# Traffic Condition Classification Model Based on Traffic-Net

**DOI:** 10.1155/2023/7812276

**Published:** 2023-01-19

**Authors:** Fengyun Cao, Sijing Chen, Jin Zhong, Yikai Gao

**Affiliations:** ^1^School of Computer Science and Technology, Hefei Normal University, Hefei 230601, Anhui, China; ^2^Hefei Xinhuo Information Technology Co., Ltd., Hefei 230000, Anhui, China

## Abstract

The classification and detection of traffic status plays a vital role in the urban smart transportation system. The classification and mastery of the traffic status at different time periods and sections will help the traffic management department to optimize road management and implement rescue in real time. Travelers can follow the traffic conditions. We choose the best route to effectively improve travel efficiency and safety. However, due to factors such as weather, time of day, lighting, and sample labeling costs, the existing classification methods are insufficient in real time and detection accuracy to meet application requirements. In order to solve this problem, this article aims to effectively transfer and apply the pretrained model learned on large-scale image data sets to small-sample road traffic data sets. By sharing common visual features, model weight parameter migration, and fine-tuning, the road is finally optimized. Traffic conditions classification is based on Traffic-Net. Experiments show that the method in this article can not only obtain a prediction accuracy of more than 96% but also can effectively reduce the model training time and meet the needs of practical applications.

## 1. Introduction

The problem of traffic congestion has become a worldwide problem. There are many factors that cause traffic congestion, including the rapid increase in the number of vehicles, insufficient rationalization of road planning, irregular driving behavior, and traffic lights. The resulting traffic delays, increased fuel consumption, and traffic accidents seriously affect people's travel safety and hinder urban development. Peak commuting, bad weather, and holiday travel are usually high periods of road congestion and secondary accidents. If we can collect enough data to accurately describe and classify urban traffic conditions and analyze the main causes of traffic congestion and secondary accidents, especially when traffic emergencies occur, rapid access to information is a key factor in organizing an optimal response; therefore, monitoring the area of the effective detection of traffic status is crucial for road traffic management. In recent years, a great deal of works have been done on traffic classification modeling, path planning, and in the broader area of transportation, where path planning algorithm [1] and energy-efficient information collection [2] are an emerging key supporting technology in the field of intelligence transportation. Many road path planning modeling and information collection methods [1–4] have been developed to understand the causes of road traffic congestion and to prevent and manage road congestion.

Our focus in this article is on the road traffic condition classification prediction using transfer learning approaches. At present, the solutions to the traffic condition classification problem can be summarized into two major categories of traffic condition classification methods that rely on the traditional manual feature representation and automatic feature extraction that rely on the deep neural network models.

Traditional image classification models mainly rely on manual feature representations, and such methods perform behavioral classification recognition by interframe difference, HOG (histogram of orientation gradients), feature back subtraction, hybrid Gaussian modeling, optical flow, and other [5, 6] feature representations and then train SVM (support vector machines) classifiers [7, 8] based on the feature representations. The support vector machine classifier approach is based on statistical learning theory with low prediction accuracy and limitations of data sample dependence and strict requirement of identical distribution for training and testing samples. Subject to the shortcomings of manual feature representation and SVM classification models, such methods cannot meet the application requirements in terms of accuracy and real-time performance.

The deep convolution neural network (DCNN) models, which combine automatic feature extraction and classification recognition, build complex neural network models in a data-driven manner with end-to-end learning mechanisms, mainly consisting of RCNN (regions with convolutional neural networks features) [9], Fast/Faster RCNN [10, 11], R-FCN (region-based fully convolutional networks) [12] as the two-stage approach based on region candidate suggestions, SSD (single shot multibox detector) [13], YOLOvX (you only look once) [14–17] for regression-based one-stage methods, and some other deep learning [18–22] methods. SSD takes VGG16 [20] as the base convolutional network architecture and adds a multiscale convolutional map for prediction result fusion of auxiliary network layer, combined with the default boxes target preselection box similar to the anchor box structure in Faster RCNN, solving the problem of different sizes of input image targets. YOLOv3 uses Darknet53 network with the introduction of residual structure as the base network, which is different from the single-level base network input of SSD, and achieves multilevel input, with higher accuracy in small target detection higher compared to SSD; although the abovementioned methods have achieved excellent results, they are limited by the lack of data samples, sample labeling, and computational resources. This type of method mainly suffers from overly complex network models that easily lead to overfitting and high false detection rates under small-sample [23–25] datasets.

To address the abovementioned problems, this article proposes a sample-based augmented traffic condition classification model. The contributions of this article can be summarized as follows:

1. The collection includes Traffic-Net dataset provided by OlafenwaMoses on GitHub, web images, and an autonomously collected image dataset Traffic-Net dataset V_HF for the created traffic condition classification, which contains four traffic categories: congested traffic, sparse traffic, accidents, and fires
2. Based on ResNet50 (residual neural network), VGG16 (Oxford Visual Geometry Group), and GoogLeNet [26] pretrained networks, migration learning is performed to fine-tune the four classifications of traffic conditions, respectively
3. The original dataset was expanded by image random geometric transformation preprocessing and CutMix sample enhancement on the basis of Traffic-Net Dataset V_HF, and the experimental results before and after enhancement were compared and analyzed

## 2. Pretraining Network Framework

The pretraining network framework is described in the following sections.

### 2.1. VGG Network Structure

**Figure 1:**
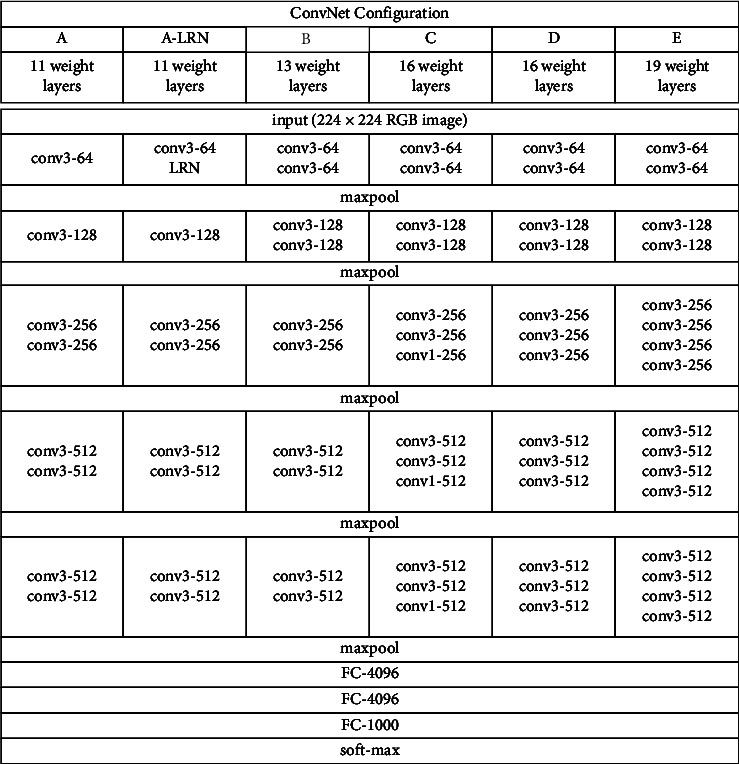
VGG model structure with different configurations [16].

In VGG, stacked small-sized convolutional kernels are used instead of larger convolutional kernels, and each convolutional layer is convolved with a modified linear unit (ReLU) as the activation function, using 3 × 3 convolutional kernels for convolution and 2 × 2 convolutional kernels for maximum pooling, so that the number of channels can be doubled, and then, the feature map can be continuously reduced, while at the same time, more nonlinear transformations in the convolutional structure of VGGNet decrease the computational effort and increase the efficiency of the CNN for image feature extraction. The VGG model in [27] has a total of six configurations with different weight layers structure, as shown in Figure 1.

### 2.2. GoogLeNet Network Structure

**Figure 2:**
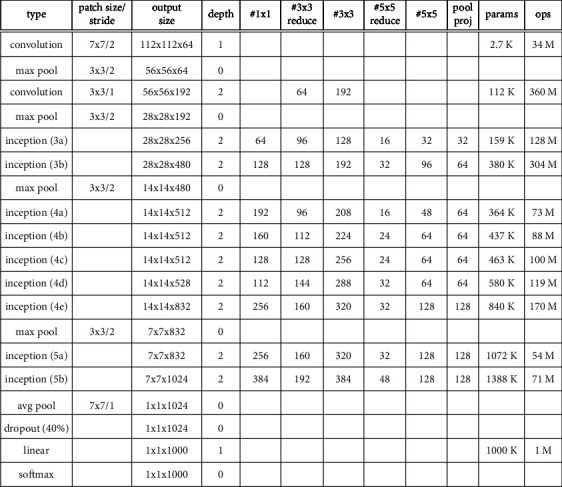
GoogLeNet Inception V1 model structure.

Feature fusion is prominent in the GoogLeNet network. Its core lies in the introduction of the Inception module, which has undergone several versions of iterative development to assemble multiple convolutional pooling operations into one module, which provides multiple kinds of convolutional kernels so that feature extraction with different sensory fields can be done and finally stitched together. Another feature of GoogLeNet is the introduction of an auxiliary classifier, which allows intermediate results to be used as the output and lets it be used with some weighting put into the final classification prediction result so that model fusion is achieved. It only works during training and is removed during prediction. The size of the original input image is 224 × 224 × 3 and is zero-averaged. The network structure of GoogLeNet Inception V1 is shown in Figure 2.

### 2.3. ResNet Network Structure

**Figure 3:**
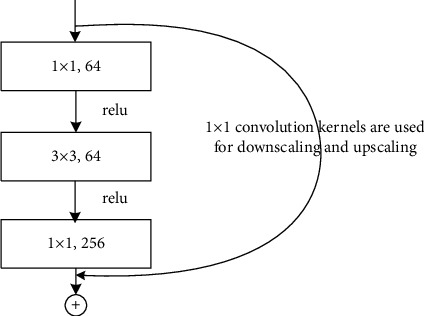
Residual block.

The residual structure of ResNet50 is shown in Figure 3. The output results are as follows:

(1) $$\begin{array}{c} H\left(X\right)=F\left(X\right)+X\text{.} \end{array}$$

When *F*(*X*) is 0, *H*(*X*) = *X* denotes the constant mapping, which is represented as a curved line in Figure 3.

**Figure 4:**
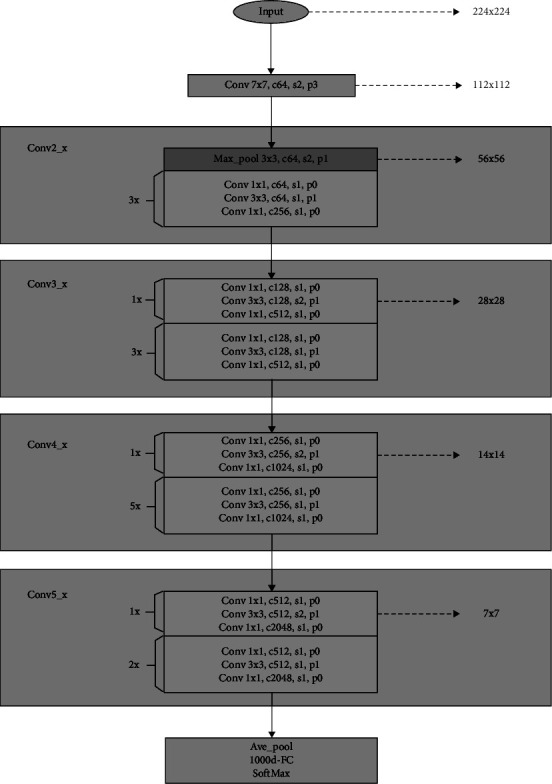
ResNet50 model structure.

The ResNet50 model has 50 layers and its structure is shown in Figure 4 ResNet50 is divided into 5 stages; each stage consists of a combination of residual structures; excluding the first stage, the next Conv2_x, Conv3_x, Conv4_x, and Conv5_x represent the remaining stages, respectively. The number of corresponding residual units are 3, 4, 6, and 3. In Figure 4, c64 means the number of channels is 64, s1 means the step size is 1, and p0 means the padding is 0. It can be seen that the ResNet50 network uses the maximum pooling to halve the size of the feature map by taking a step size of 2 except for the first stage, and the other stages use the convolution operation by taking a step size of 2 to achieve the same effect.

## 3. Traffic Condition Classification Based on Pretrained Network Model Migration

Although there are many reasons for different feature distributions in different datasets [17], it is not difficult to find that shallow networks can extract low-level features such as edges and contours. As the layers of the network deepen many local features will be formed, and then, they are combined to form the whole. Due to the following similarities, similar data, similar tasks, and similar models, it then becomes possible to take a model trained in an old domain and apply it to a new domain [18], and this process is called migration learning. Due to the large and complex structure of the deep neural networks, designing and testing models are expensive and time-consuming, and one of the convenient and effective ways to improve the efficiency of model training (especially when the number of samples is small) can be done by migration learning techniques. Generally, migration learning methods using pretrained models are divided into feature extraction and fine-tuning, which can be chosen according to the sample size and characteristics of a particular application area. In this article, we choose a migration learning method based on fine-tuning of parameters to improve the training speed and recognition rate of pretrained models on domain-specific sample datasets by freezing the shallow weights and fine-tuning the retraining for the deeper networks. The features learned in the convolutional layer are generalizable to different samples, especially in the shallow network layer because the shallow convolutional network layer learns local subtle features, while the deep layer is more biased to local or global object contour features. The parameter fine-tuning mechanism also effectively avoids overfitting due to the small number of samples, which leads to overly complex model parameters.

In this article, three pretrained models are selected for multistrategy fine-tuning experiments of weight parameters in MATLAB2021a environment. The parameter fine-tuning strategies mainly include freezing the weights of shallow layers, relearning the weights, and freezing all weights of the fully connected layers. Algorithm 1 outlines the flowchart of the proposed method.

### 3.1. Traffic Condition Type and Sample Construction

**Figure 5:**
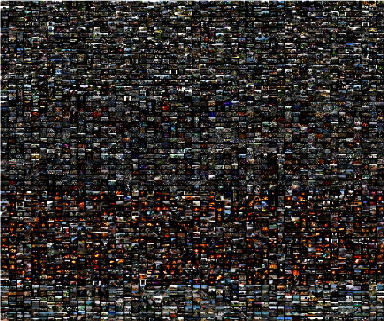
Dataset display.

The experimental dataset Traffic-Net Dataset V_HF consists of three main parts: (1) Traffic-Net Dataset V1 provided by OlafenwaMoses on GitHub [28]; (2) images of the same type on the network; (3) images collected from traffic intersections. Four traffic categories are included: congested traffic, sparse traffic, accidents, and accidental fires. Each category has 2,000 images, of which 1,400 are used for training and 600 for testing. In this experiment, each category is placed under a folder named after the corresponding category, and the training data are divided into two parts: 70% of the training set and 30% of the validation set, and the dataset images are displayed on MATLAB using the montage () function as shown in Figure 5. The training set (train) is used to train the model, the validation set is used to check whether the hyperparameter adjustment is effective, and the performance of the model is evaluated by the test set.

### 3.2. Data Preprocessing

The diversity of data collection brings image data in the dataset with different resolutions. Using the constructed dataset for network training, the input image must match the input size of the network model, and according to the data situation and the classification target, the sample data can be preprocessed and sample enhancement operation, and the image preprocessing can greatly improve the algorithm prediction accuracy.

#### 3.2.1. Image Random Geometric Transformation

This includes image resizing, random rotation, random cropping, random panning, color transformation, and other preprocessing operations, which result in different data in each round due to the random nature of the operation. For example, for the same image in some rounds flipped and some rounds not flipped, so the data used for training in each round are different, and the purpose of sample enhancement is achieved.

#### 3.2.2. CutMix Sample Enhancement

CutMix generates a new training sample $\left(\tilde{x},\tilde{y}\right)$ by combining two training samples (*x*_*a*_,*y*_*a*_) and (*x*_*b*_,*y*_*b*_); the new sample is used to train the network model with the original loss function. The sample combination operation is defined as shown in the following equation:

(2) $$\begin{array}{c} \tilde{x}=M\;☉\;x_{a}+\left(1-M\right)\;☉\;x_{b}\text{,}\\ \tilde{y}=\lambda y_{a}+\left(1-\lambda \right)y_{b}\text{,} \end{array}$$

where *M* ∈ {0,1}^*W*×*H*^ denotes the marker mask for the image cropping and retention region, the filled region is 1 and the rest is 0, ☉ representing pixel-by-pixel, *λ* belonging to Beta(*∂*, *∂*), and usually set to 1 in experiments *∂*, i.e., *λ* obeying a uniform distribution of (0, 1).

## 4. Experimental Results

The experimental results are described in the following sections.

### 4.1. Experimental Setup

**Figure 6:**
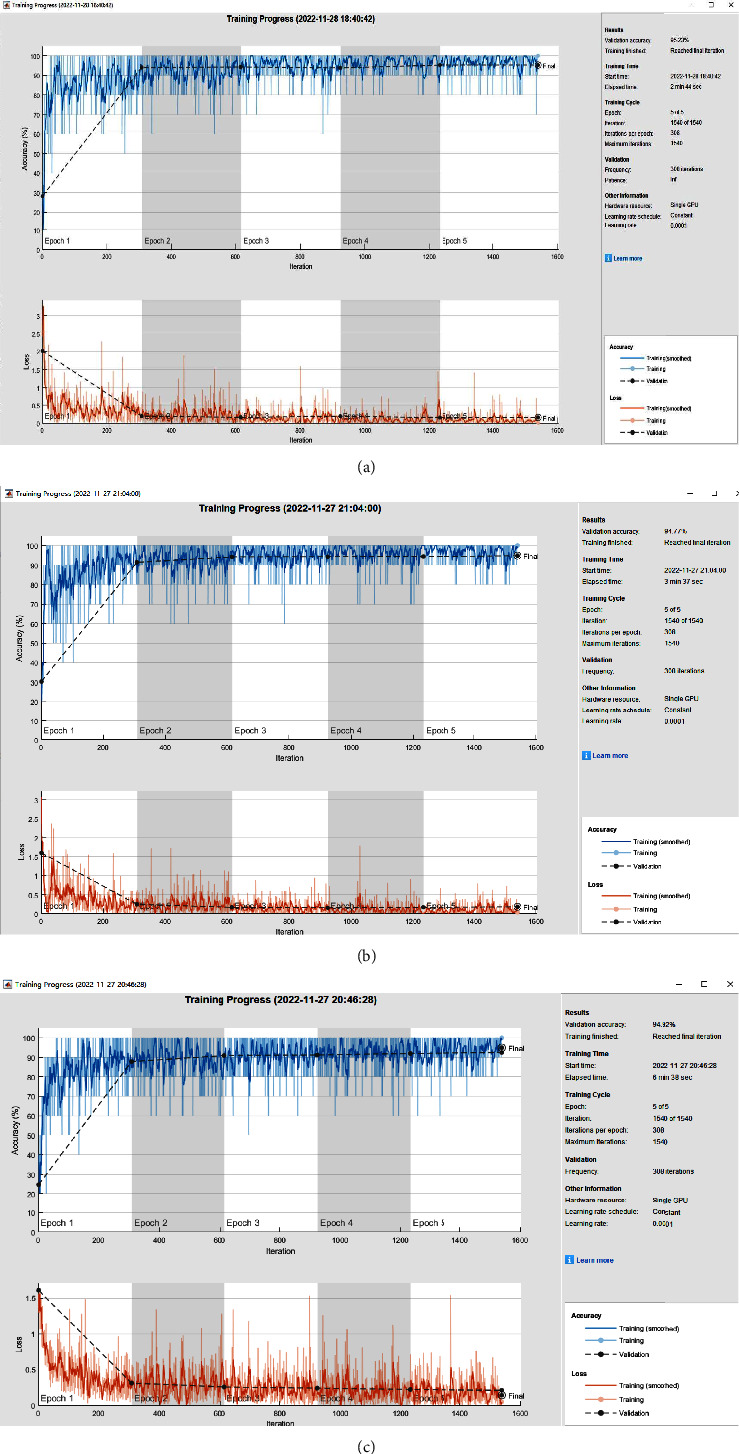
Training progress. (a) VGG16 pretrained model. (b) GoogLeNet pretrained model. (c) Resnet50 pretrained model.

The hardware environment for the experiments in this article is as follows: NVIDIA GeForce GTX 2080TI GPU with 11G video memory and Intel(R) Core (TM) i7-9700 CPU @ 3.00 GHz 3.00 GHz workstation; software environment: MATLAB2021a; hyperparameter settings: initial learning rate is set to 0.0001; using SGDM as the network optimization algorithm, we replace the fully connected layer of the pretrained network to modify the classification category and freeze the remaining layer weight parameters before retraining on the Traffic-Net Dataset V1 dataset; VGG16, GoogLeNet, and ResNet50 are the three pretrained models in the case of MaxEpochs = 5 and MiniBatchSize = 10. The validation set accuracy and training elapsed time are shown in Table 1, and the training process is shown in Figures 6(a)–6(c).

Comparing the training process time and validation accuracy of each model, it is easy to find that ResNet50 takes the longest time to train when the three models achieve close accuracy.

### 4.2. Sample Augmentation Control Experiment Results

**Figure 7:**
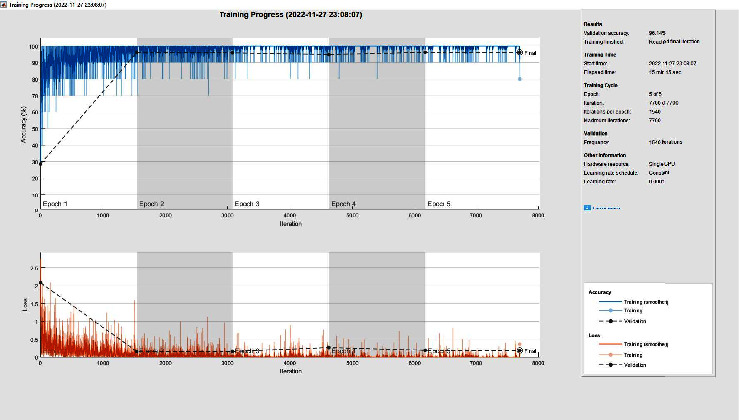
Sample enhancement training process.

Sample augmentation mainly includes the following. (1) The data augmentation operation comes with the deep learning training in MATLAB, by randomly rotating, panning, and resizing the training samples in each iteration of training, so that the sample data are different in each round of training to achieve sample augmentation. (2)The number of samples is increased by collecting the same type of image data on the network. However, the downloaded photos cannot be converted to the same dimension due to their different formats, so they are converted to grayscale images while adding images. The “ColorPreprocessing” option in MatLab is used to ensure that all enhanced images have the same number of channels. (3) Using CutMix to select images from the samples, local areas of the images are Cropping and are used to superimpose local areas of the image onto other sample images, and new training samples are generated to enhance the dataset. As shown in Figure 7, the final validation accuracy reached 96.14% using the GoogLeNet pretrained model, which shows that the larger the dataset is, the higher the validation accuracy is.

### 4.3. Experimental Results of Hyperparameter Settings

The experimental results of hyperparameter settings are explained in the following sections.

#### 4.3.1. Effect of Learning Rate Setting on Model Accuracy

With MaxEpochs = 5, MiniBatchSize = 8, and the optimizer as SGDM, setting the learning rate as 0.001, 0.0003, 0.0001, and 0.00001, respectively, the accuracy of the validation set obtained by using the GoogLeNet pretrained model is 93.70%, 94.35%, 94.77%, and 87.78%. It can be seen in Table 2 that the model validation accuracy is strongly influenced by the initial learning rate when using the SGDM optimizer.

#### 4.3.2. Optimizer

Keeping other parameters unchanged and changing only the model optimizer settings, Adam's squared gradient decrement factor is set to 0.99 and the accuracy of the validation set using the GoogLeNet pretrained model is 89.91%, but the accuracy using SGDM is 94.35%.

#### 4.3.3. Number of Training Rounds

**Figure 8:**
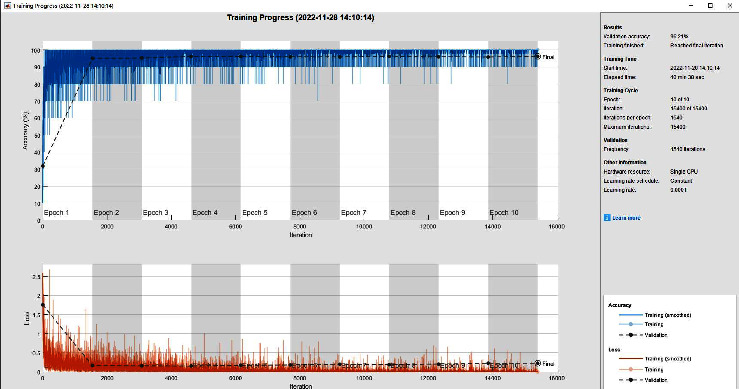
Super parameter modification comparison training process.

Keeping the other parameters unchanged and changing only the number of training rounds and MaxEpochs from 6 to 10, the accuracy of the validation set using the GoogLeNet pretraining model is changed from 95.53% to 96.21%, respectively. From the results of the training process in Figure 8, we can see that increasing the number of training rounds does not change the model performance when the model reaches convergence but only increases the training time.

## 5. Analysis and Discussion

The analysis and discussion are described in the following sections.

### 5.1. Model Evaluation

**Figure 9:**
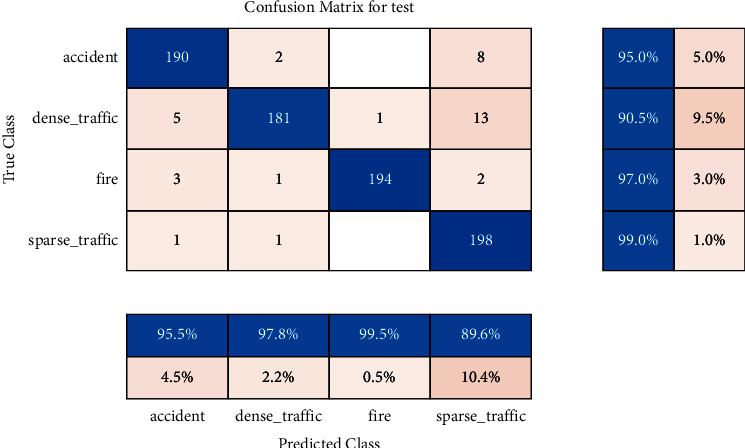
Confusion matrix for test.

The trained model is tested on the test set, and the confusion matrix is drawn as shown in Figure 9, where the correct predictions are distributed on the diagonal, and the rows and columns also show the recall and accuracy rates for each class, respectively. Recall, also known as the full rate, is shown in Figure 9, where the denominator is the sum of the rows and the numerator is the correct prediction for each class. Precision, also called accuracy, is shown in Figure 9, where the denominator is the sum of the columns and the correct prediction of each class is the numerator. Their numerators are the same, but the denominators are different.

We calculate precision and recall of the integrative index F1-measure, as shown in the following equation:

(3) $$\begin{array}{c} F1-measure=\frac{2}{\left(1/Precision\right)+\left(1/Recall\right)}\text{.} \end{array}$$

**Figure 10:**
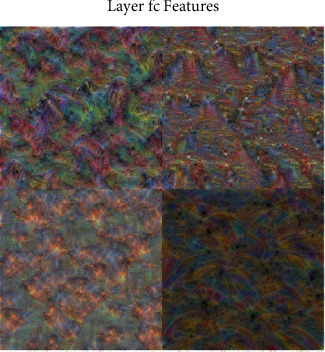
Visual full connection layer.

The value of F1-measure is distributed between (0, 1), and the closer to 1, the better. Its value is calculated from Figure 9 as 94.67%, which shows that the performance of the model is good. It can also be visualized by visualizing the intermediate network layers. Selecting the fully connected layer, a detailed image of each classification with strong activation was generated as shown in Figure 10. The image generated for the “fire” category contains obvious fire color features.

### 5.2. Error Analysis

**Figure 11:**
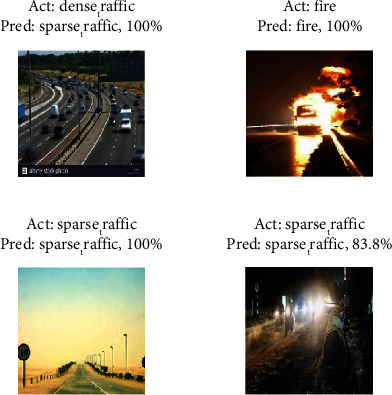
Examples of mispredictions.

An example of one prediction result error is shown in the first panel on the left of Figure 11, where the model incorrectly predicts congested traffic as sparse traffic.

**Figure 12:**
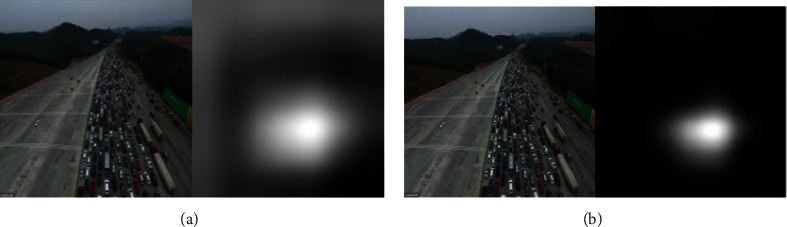
Visual error prediction. (a) Strongest activation region for incorrect prediction. (b) The strongest activation area incorrectly predicted after ReLU function activation.

The error example arises when the model has no evaluation criteria for the number of vehicles in sparse traffic and predicts sparse traffic when the extracted feature is an empty road. This also reflects the problem of how to solve this problem when the model's region of interest is not focused on the correct category. Another reason for the error is that the image is not captured with a clear vision, resulting in no texture; then, the convolutional layer does not extract features, and the congested traffic is discriminated as sparse traffic. The visualization error prediction of the inception_5a-5 × 5 convolutional layer with the strongest activation channel is shown in Figure 12(a), and it can be seen that the off-white pixels correspond to the original image in addition to the vehicle part being activated; there are distant forests and open roads. The image after the ReLU activation is shown in Figure 12(b), and it can be seen that only the vehicle part of the off-white is activated.

**Figure 13:**
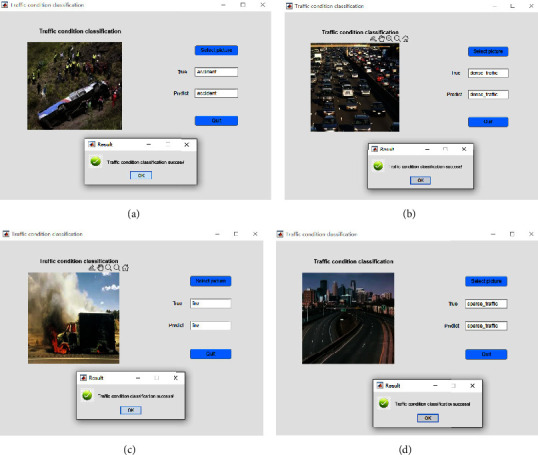
Classification test results. (a) Traffic accident classification results. (b) Classification results of dense traffic. (c) Classification results of fire traffic. (d) Classification results of sparse traffic.

For better application deployment, the algorithm classification test results are displayed in combination with the UI interface, as shown in Figures 13(a)–13(d).

## 6. Conclusion

In this article, we designed and implemented a traffic classification model based on migration learning on the basis of the Traffic-Net V1 dataset and conducted a multidimensional comparative analysis of various deep learning frameworks with multiple strategies such as sample data enhancement and fine-tuning parameters to improve the model, and the experimental comparison results showed that the model migration has good generalization ability, and the classification recognition is applied on the dataset of the target domain. The accuracy rate reaches more than 95%, which is well adapted to the classification recognition task in the target domain.

## Figures and Tables

**Algorithm 1:**
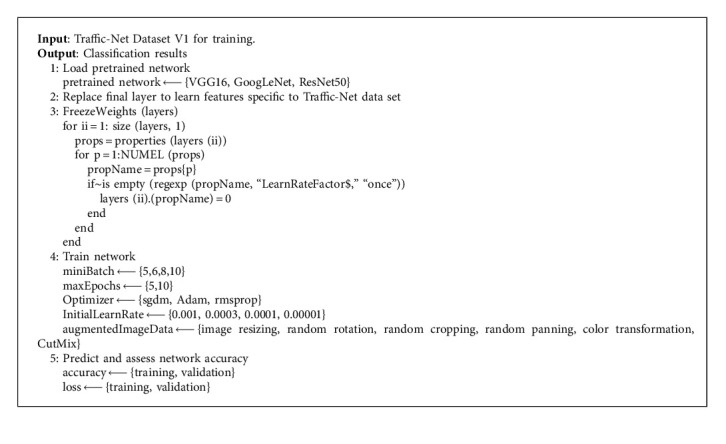
Transfer learning model algorithm.

**Table 1:** Comparison of training process time.

Pretrained models	Accuracy (%)	Time-consuming training (min)
VGG16	95.23	2.44
GoogLeNet	94.77	3.37
Resnet50	94.92	6.38

**Table 2:** Comparison of different learning rates.

Accuracy (%)	Learning rates
93.70	0.001
94.35	0.0003
92.13	0.0001
87.78	0.00001

## Data Availability

The data used to support the findings of the study can be obtained from the corresponding author upon request.
